# Supplementary material for: The impact of ethical implications intertwined with tuberculosis household contact investigation: A qualitative study
Source: PLoS One. 2026 Mar 30;21(3):e0306848. doi: 10.1371/journal.pone.0306848 (PMC13035131; doi:10.1371/journal.pone.0306848)
Supplement: S1 Table — (DOCX) [file pone.0306848.s001.docx]

**S1 Table.**

| **AGE (years)** | **GENDER** | **HIV STATUS** | **DISTRICT** | **PARTICIPANT TYPE** |
| --- | --- | --- | --- | --- |
| **In-depth Interviews** | | | | |
| 43 | M | Negative | Soshanguve | Person with TB |
| 29 | F | Positive | Soshanguve | Person with TB |
| 46 | F | Positive | Soshanguve | Person with TB |
| 61 | M | Positive | Soshanguve | Person with TB |
| 22 | M | Negative | Vhembe | Person with TB |
| 28 | F | Positive | Vhembe | Person with TB |
| 40 | F | Positive | Vhembe | Person with TB |
| 25 | M | Negative | Vhembe | Person with TB |
| 68 | F | Negative | Capricorn | Person with TB |
| 37 | M | Negative | Capricorn | Person with TB |
| 26 | F | Negative | Capricorn | Person with TB |
| 26 | M | Negative | Capricorn | Person with TB |
| 32 | F | Positive | Soshanguve | Household member |
| 39 | F | Positive | Soshanguve | Household member |
| 67 | M | Positive | Soshanguve | Household member |
| 39 | M | Positive | Soshanguve | Household member |
| 88 | F | Unknown | Vhembe | Household member |
| 54 | F | Negative | Vhembe | Household member |
| 33 | F | Negative | Vhembe | Household member |
| 32 | F | Positive | Vhembe | Household member |
| 58 | F | Negative | Capricorn | Household member |
| 30 | F | Negative | Capricorn | Household member |
| 81 | M | Unknown | Capricorn | Household member |
| 21 | M | Negative | Capricorn | Household member |
| **Focus Group Discussions** | | | | |
| 62 | F | Positive | Soshanguve | Person with TB |
| 58 | M | Positive | Soshanguve | Person with TB |
| 64 | M | Positive | Soshanguve | Person with TB |
| 52 | M | Positive | Soshanguve | Person with TB |
| 34 | M | Negative | Soshanguve | Person with TB |
| 78 | M | Positive | Soshanguve | Person with TB |
| 36 | M | Positive | Vhembe | Person with TB |
| 43 | M | Negative | Vhembe | Person with TB |
| 47 | M | Positive | Vhembe | Person with TB |
| 19 | F | Unknown | Vhembe | Person with TB |
| 47 | F | Positive | Capricorn | Person with TB |
| 45 | M | Negative | Capricorn | Person with TB |
| 30 | M | Negative | Capricorn | Person with TB |
| 35 | M | Negative | Capricorn | Person with TB |
| 29 | M | Negative | Capricorn | Person with TB |
| 32 | F | Positive | Soshanguve | Household member |
| 45 | M | Positive | Soshanguve | Household member |
| 58 | F | Positive | Soshanguve | Household member |
| 49 | F | Positive | Soshanguve | Household member |
| 71 | F | Positive | Soshanguve | Household member |
| 48 | F | Positive | Soshanguve | Household member |
| 62 | F | Positive | Soshanguve | Household member |
| 20 | M | Negative | Vhembe | Household member |
| 72 | M | Unknown | Vhembe | Household member |
| 21 | F | Unknown | Vhembe | Household member |
| 35 | M | Unknown | Vhembe | Household member |
| 22 | F | Positive | Vhembe | Household member |
| 64 | M | Positive | Vhembe | Household member |
| 66 | F | Unknown | Vhembe | Household member |
| 21 | F | Negative | Vhembe | Household member |
| 25 | F | Positive | Vhembe | Household member |
| 42 | M | Positive | Capricorn | Household member |
| 70 | F | Positive | Capricorn | Household member |
| 62 | M | Positive | Capricorn | Household member |
| 43 | F | Positive | Capricorn | Household member |
| 27 | F | Positive | Capricorn | Household member |
| 35 | F | Negative | Capricorn | Household member |
| 27 | M | Negative | Capricorn | Household member |
| 23 | F | Positive | Capricorn | Household member |
