# Supplementary material for: The impact of ethical implications intertwined with tuberculosis household contact investigation: A qualitative study
Source: PLoS One. 2026 Mar 30;21(3):e0306848. doi: 10.1371/journal.pone.0306848 (PMC13035131; doi:10.1371/journal.pone.0306848)
Supplement: S1 File — (DOCX) [file pone.0306848.s002.docx]

**S1 File.**

**Ethics of household contact investigation in South Africa: interview guide**

**Kharituwe Bioethics Supplement**

**In-depth Interview Guide Version 1.0/ 13 October 2020**

**Introduction:**

*Thank you for being here today. I want to remind you before we start talking, there are no right or wrong answers. I am here to learn from you and hear your ideas and suggestions.*

**Open-ended questions:**

*As you know, today we are going to speak about tuberculosis (TB), which is an infectious disease that mainly affects the lungs. Often people with TB have a cough, fever or weight loss – sometimes they may have TB for months or even years without knowing it. TB is spread from one person to another through the air. Because people often spend time with their household members, if one household member has TB and is coughing, other household members may also get TB. There is a cure for TB, and testing and treatment are free at the clinic.*

*One of the ways that is often used to find more people with TB and tell them about their treatment options is for health workers to visit the household of a person with a recent diagnosis of TB and check the other people who live in the household for TB. This may involve asking people if they have any of the symptoms of TB and taking a sputum sample from people who have TB symptoms. This way of finding new cases of TB is called household contact investigation. Often household members are also asked if they would like to be tested for HIV at these household visits.*

*Let’s start with getting some of your thoughts about household visits to check people for TB.*

1. I’d like to first start by asking what you think of health workers visit to the households of people who have TB to check other household members for TB?

Probe: Do you think it is important for health workers to visit the households of people who have TB to check other household members for TB?

Probe: Please explain to me whether you think visiting the households of people who have TB to check household members for TB is a good or bad way to find new cases of TB?

Probe: What would you propose as a different way to finding new cases of TB other than healthcare workers visiting homes to check household members for TB?

Probe: What are the benefits of visiting the households of people who have TB to check household members for TB?

Probe: What are some things that might not be good about visiting the households of people who have TB to check household members for TB?

Probe: If health workers do not visit the households of people who have TB to check their household members, what might happen?

1. [For index cases only]: Can you tell me how you felt or would feel about having someone come to your house to check other household members for TB?

[For contacts only]: If or when someone in your household was diagnosed with TB, can you tell me how you felt or would feel about having someone come to your house to check you and other household members for TB?

Probe: Do you think everyone in your household would feel comfortable having someone come to the house to check household members for TB? Why or why not? Which people would or would not feel comfortable with this, and why?

Probe: Please describe any concerns you would have about having someone come to the house to check household members for TB?

Probe: What are the reasons that people may or may not want to be checked for TB in the household?

1. I would like to hear your thoughts about, when a health worker comes to the household of someone recently diagnosed with TB to check other household members for TB, how that health worker should explain the reason for the visit. What do you think the health worker should say to the household members about the reason for their visit, and why?

Probe: Please tell me about the good or the bad things about the health worker saying the reason for the visit is because someone in the household has been diagnosed with TB and invited us to come.

Probe: Please tell me about the good or the bad things about the health worker saying the reason for the visit is because they are trying to test for TB in selected households in this community.

1. [Index case only]: Did you tell your household members that you have TB?? Why or why not?

Probe: (If yes to Question 4) Please tell me about your experience telling your household members that you were diagnosed with TB.

Probe [index case only]: Please describe anything that worried you about telling your household members that you had TB? Were there any household members who you decided not to tell? Why did you not want to tell these people?

Probe [index case only, if they did disclose]: Please describe how household members reacted when you told them about your TB diagnosis.

[Contact only]: Were you told by anyone that a member of your household was diagnosed with TB? [If yes,] please tell me about your experience finding out that your household member had TB. [If no,] if someone in your household was diagnosed with TB, would you want to know – and if so, how would you like to be told? Please explain why.

Probe [contact only]: How did or would you feel when you found out or if you were to find out your household member had TB?

Probe [contact only]: How did or would you react when you found out or if you were to find out your household member had TB?

Probe [contact only]: Please describe any worries or concerns you had or would have when you found out or if you were to find out your household member had TB?

1. [Index case only]: Please tell me about the reasons for your decision to tell your household members that you had been diagnosed with TB?

Probe: Please describe to me if you had in mind the health of your household members when thinking about whether to tell them about your TB diagnosis.

Probe: Please tell me about anything that made you not want to tell your household members about your TB diagnosis.

Probe: Please tell me about how knowing that health care workers might come to your house influenced your decision of whether or not to tell your household members about your TB status.

1. [Index case only]: I would like to know more about your experience after you were diagnosed with TB. In general, how do you feel you are treated in your community because of your TB diagnosis?

Probe: Do your family members know about your diagnosis? How are you (would you be) treated by your family after learning of this diagnosis?

Probe: Do your close neighbors know about your diagnosis? How are you (would you be) treated by neighbors after learning of this diagnosis?

Probe: Do your close friends know about your diagnosis? How are you (would you be) treated by friends after learning of this diagnosis?

1. Please tell me about how you think having someone come to the house to check household members for TB might impact how you are treated in your community?

Probe: If people in your community notice someone is coming to the house to check for TB, please describe whether they might treat you differently?

Probe: Do you think their reaction might be different if they were visited by people in plain clothes and unmarked cars versus in uniforms and marked cars?

Probe: How do people in this community think about someone’s TB status in relationship to their HIV status? If they know that someone has been diagnosed with TB, will they assume that person has HIV as well?

Probe: Please describe how you think people in this community feel about someone who has TB?

1. Do you think people in this community would discuss their TB diagnosis with others? Why or why not?

Probe: What are the reasons someone might not want to tell others in the community that they have been diagnosed with TB?

*For the final part of our discussion, I am going to ask you about different timings of household contact investigation. Each involves having a health worker visit the household, but the timing of these visits are different in the three types. As I mentioned earlier, when I say household contact investigation I mean when health workers visit the household of a person with a recent diagnosis of TB and check the other people who live in the household for TB, and usually offer HIV testing as well. We are interested in your thoughts.*

*The first time that health workers could come and visit households after someone was diagnosed with TB is during normal business hours (9:00-17:00). This is what is currently done in places where TB contact investigation is done. A different time when health workers could visit instead would be in the evenings and on the weekends, when some people might be more likely to be home. And another possibility for timing, instead, is to visit on the holidays like the festive season or Easter. This holiday approach might have the benefit of having large families home during those times, but would also potentially involve delays (for example, if someone were diagnosed with TB in July and couldn’t be visited until December). Do you have any questions about these types of household visits before we go further?*

*Now let’s talk in more detail about each of these types of household visits…*

Note: Ask the following questions for each type of household visit (holiday, off-peak, and routine). Remind of participant of the definition for each type of household visit.

1. What do think about this type of visit to households of people who have TB to check other household members for TB?

Probe: What are the benefits of this type of visit to households of people who have TB to check other household members for TB?

Probe: What are the things that might not be good about this type of visit to households of people who have TB to check other household members for TB?

Probe: Please describe anything that would make you uncomfortable about this type of visit to households of people who have TB to check other household members for TB.

Probe: Please describe anything that would be good about this type of visit to households of people who have TB to check other household members for TB.

1. What do you think about offering HIV testing to others in the household when visiting the household during this type of visit to check for TB?

Probe: Please describe for me any problems there might be if health workers offer HIV testing at the same time as they come to check household members for TB.

Probe: Please tell me about anything that you think is good about having HIV testing offered to household members at the same time they are being checked for TB.

1. Thinking about all three types of household visits – holiday, off-peak, and routine – which approach do you think is best, and why?

Probe: Tell me about your choice…what would make that the best type over the others?

*Thank you for your time…this information has been very valuable.*
