# Supplementary material for: The impact of ethical implications intertwined with tuberculosis household contact investigation: A qualitative study
Source: PLoS One. 2026 Mar 30;21(3):e0306848. doi: 10.1371/journal.pone.0306848 (PMC13035131; doi:10.1371/journal.pone.0306848)
Supplement: S2 File — (DOCX) [file pone.0306848.s003.docx]

**S2 File.**

**Ethics of household contact investigation in South Africa: focus group discussion guide**

**Kharituwe Bioethics Supplement**

**Focus Group Discussion Guide Version 1.0/ 13 October 2020**

**Introduction:**

*Thank you all for being here today. I want to remind everyone before we start talking that there are no right or wrong answers. We are here to learn from you and hear your ideas and suggestions. We want all of you to tell us what you think and learn from you and your experiences. ______________________ will lead the discussion today by asking a question to the whole group and call on people to get their thoughts. We want to hear from everyone. __________________ will be taking notes while we talk.*

*Does anyone have any questions before we start?*

**Open-ended questions:**

*As you know, today we are going to speak about tuberculosis (TB), which is an infectious disease that mainly affects the lungs. Often people with TB have a cough, fever or weight loss – sometimes they may have TB for months or even years without knowing it. TB is spread from one person to another through the air. Because people often spend time with their household members, if one household member has TB and is coughing, other household members may also get TB. There is a cure for TB, and testing and treatment are free at the clinic.*

*One of the ways that is often used to find more people with TB and let them know about it is for health workers to visit the household of a person with a recent diagnosis of TB and check the other people who live in the household for TB. This may involve asking people if they have any of the symptoms of TB and taking a sputum sample from people who have TB symptoms. This way of finding new cases of TB is called household contact investigation. Often household members are also asked if they would like to be tested for HIV at these household visits.*

*Let’s start with getting some of your thoughts about household visits to check people for TB.*

1. As members of this community, please tell me what you think about having health workers visit the households of people who have TB?

Probe: Do you think it is important to have health workers visit the households of people who have TB? Why or why not?

Probe: Tell me about why you think this might be good for the households who are visited?

Probe: Tell me about why you think this might be good for the community?

Probe: How do you think having a health worker visit the household would make people feel?

Probe: Do you think people might feel pressured into doing something they don’t want to do? Why?

1. Thinking about all the benefits and negatives, please tell me about if you think that visiting households of people who are diagnosed with TB is something that should be done?

Probe: Do you think it’s something that clinics should focus on doing? Why or why not?

1. *I am going to present a scenario and I would like you all to tell me your thoughts about it:* Please tell me what you think about a scenario where there is a household and the people who stay there want to be checked for TB, but the person in the household who has been diagnosed with TB decides they do not want the health workers to visit the household to check everyone for TB.

Probe: What are the positive and negatives of someone with TB deciding that their household cannot be visited and other household members checked for TB?

Probe: In your opinion, is it fair or unfair for the person with TB to make this decision, and why?

1. *I am going to present another scenario and I would like you all to tell me your thoughts about it:* Please tell me what you think about a scenario where there is someone in the household who has been diagnosed with TB, and only some of the family members want to be checked for TB, but others do not want to?

Probe: Should the healthcare workers who are visiting ask people if they want to get tested for TB or tell them that they should get tested?

Probe: Please tell me your thoughts on the responsibility of household members who have had contact with the person who has been diagnosed with TB to get checked?

1. How does your community view people from outside of the community coming to someone’s house?

Probe: How do people in the community react or act when people from outside the community come to someone’s house?

1. Please tell me about how people in the community treat or act towards someone who they know has been diagnosed with TB.

Probe: Describe for me how people in the community treat or act towards someone they know has TB compared to someone who does not.

Probe: Describe for me how people in the community act towards household members of someone who has TB.

1. Do you agree or disagree that people in this community make assumptions about the HIV status of someone who has been diagnosed with TB or the HIV status of the household where someone has TB? Please explain.

Probe: What do people in the community assume about someone’s HIV status if they are diagnosed with TB, and why?

1. As members of this community, what do you think about offering HIV tests to all household members, when coming to visit a household for TB testing because a household member has TB?

Probe: What are the things that are good about offering HIV tests to all household members?

Probe: What are the bad things or negatives about offering HIV tests to all household members?

1. Please tell me your thoughts on how household visits to check for TB among household members could be made less shameful to patients and household members?

Probe: Tell me about things that the person or people visiting the home could do to not cause households to be stigmatized by individuals or the community?

*For the final part of our discussion, I am going to ask you about different timings of household contact investigation. Each involves having a health worker visit the household, but the timing of these visits are different in the three types. As I mentioned earlier, when I say household contact investigation I mean when health workers visit the household of a person with a recent diagnosis of TB and check the other people who live in the household for TB, and usually offer HIV testing as well. We are interested in your thoughts.*

*The first time that health workers could come and visit households after someone was diagnosed with TB is during normal business hours (9:00-17:00). This is what is currently done in places where TB contact investigation is done. The second time that health workers could visit would be in the evenings and on the weekends, when some people might be more likely to be home. The third time is on the holidays like the festive season or Easter. This approach might have the benefit of having large families home during those times, but would also potentially involve delays (for example, if someone were diagnosed with TB in July and couldn’t be visited until December). Do you have any questions about these types of household visits before we go further?*

Note: Make use of a flip chart to note the positives and negatives of each strategy. Reference back to the flip chart to remind people of each type of contact investigation for questions asking them to compare all three.

*Now let’s talk in more detail about each of these different types of timing…we’ll start with the holiday one.*

1. How do you think this type of visit of going to the houses of people with TB to check others in the house for TB on the holidays will be accepted by this community?

Probe: Would people in this community be willing to have someone come to their household during the holidays to check for TB, and why or why not?

Probe: What concerns might people in this community have about health workers going to the houses of people with TB to check others in the house for TB on the holidays?

1. What are the things that people in this community might like about this type of visit?

Probe: What are the benefits?

1. What are some of the things that people in this community might not like about this type of visit to check people in the household for TB?

Probe: What are the things that might not be good about this type of visit during the holidays?

*Next let’s talk about the off-peak timing [remind of definition].*

1. How do you think going to the houses of people with TB to check others in the house for TB on the evenings and weekends will be accepted by this community?

Probe: Would people in this community be willing to have someone come to their household evenings and weekends to check for TB, and why or why not?

Probe: What concerns might people in this community have about a health worker going to the houses of people with TB to check others in the house for TB on evenings and weekends?

1. What are the things that people in this community might like about this type of visit to check people in the household for TB?

Probe: What are the benefits?

1. What are some of the things that people in this community might not like about this type of visit to check people in the household for TB?

Probe: What are the things that might not be good about this type of visit during evenings or weekends?

*Moving on to the routine type of visit [remind of definition].*

1. How do you think the routine type of visit is accepted by this community?

Probe: Are people in this community be willing to have someone come to their household during normal business hours to check for TB, and why or why not?

Probe: What concerns do you think people in this community have about this type of visit to check people in the household for TB?

1. Thinking about all three types of timing for household visits – holiday, off-peak, and routine – which approach would you choose as the best for your community, and why?

Probe: Tell me about your choice…what would make that the best type of timing over the others?

*Thank you all for your time…this information has been very valuable.*
